# Two new species of *Phyllanthus* (Phyllanthaceae) from Thailand

**DOI:** 10.3897/phytokeys.136.47625

**Published:** 2019-12-11

**Authors:** Pimwadee Pornpongrungrueng, Pranom Chantaranothai, John A.N. Parnell, Trevor R. Hodkinson

**Affiliations:** 1 Applied Taxonomic Research Center, Department of Biology, Faculty of Science, Khon Kaen University, Khon Kaen 40002, Thailand Khon Kaen University Khon Kaen Thailand; 2 Herbarium, Department of Botany, School of Natural Sciences, and Trinity Centre for Biodiversity Research, Trinity College Dublin, the University of Dublin, Dublin 2, Ireland University of Dublin Dublin Ireland

**Keywords:** diversity, Euphorbiaceae, new taxa, revision, taxonomy

## Abstract

Two *Phyllanthus* species are newly described from a limestone mountain in the north of Thailand. The first species, *P. huamotensis* Pornp., Chantar. & J.Parn., **sp. nov.**, is one of the most distinct *Phyllanthus* species easily distinguished by its reddish branchlets and stem, conspicuous reddish venation, especially on the lower leaf surface, red sepals with long fimbriate margin and red capsule with papillose-puberulous surface. The second species, *P. chantaranothaii* Pornp., J.Parn. & Hodk., **sp. nov.**, is similar to *P. pulcher* Wall. ex Müll.Arg., but it is distinguished by its puberulous upper leaf surface and pistillate flowers which have red, narrowly lanceolate sepals with a white, long fimbriate margin, puberulous outer side as well as puberulous pedicel.

## Introduction

*Phyllanthus* L. (Phyllanthaceae) is mainly distributed in tropical and subtropical regions (Radcliffe-Smith 2001; Webster 2014). The classification of *Phyllanthus* is still contentious (Kawakita and Kato 2017) because molecular phylogenetic studies have indicated that *Phyllanthus*, as previously circumscribed, was not monophyletic (Kathriarachchi et al. 2006). Therefore, some authors suggested merging other closely related genera in *Phyllanthus*, such as *Breynia* J.R.Forst. & G.Forst., *Glochidion* J.R.Forst. & G.Forst. and *Sauropus* Blume (Hoffmann et al. 2006), while others suggested division of *Phyllanthus* into several monophyletic and morphologically recognisable genera (Pruesapan et al. 2012; van Welzen et al. 2014). The most recent publication by Bouman et al. (2018) recorded 880 species of *Phyllanthus* and, amongst these, they were able to place 837 species in 18 subgenera, 70 sections and 14 subsections; 43 species remained unassigned. *Phyllanthus* is, therefore, one of the most diverse genera of flowering plants and its species often have a high degree of endemism. For example, in China, there are about 32 species reported, 13 of which are endemic (Li and Gilbert 2008). Thirty-six species of *Phyllanthus* s.str. (excluding *Breynia*, *Glochidion* and *Sauropus*) were reported for the Flora of Thailand by Chantaranothai (2007). Lately, two more new species have been included by Kantachot and Chantaranothai (2013) and Pornpongrungrueng et al. (2017). Thus, the total Thai species number has increased to 38, five of which are endemic to Thailand. Recently, two *Phyllanthus* taxa were discovered in Umpang district, Tak province, in the northern part of Thailand; they were investigated and are described herein as new species.

## Methods

Field collections and herbarium specimens from various herbaria, as well as taxonomic literature, were examined. The herbarium abbreviations follow Index Herbariorum (Thiers 2019, continuously updated). The morphological descriptions and measurements were taken from dried specimens.

## Results

### Taxonomic treatment

#### Phyllanthus huamotensis

Taxon classificationPlantaeMalpighialesPhyllanthaceae

Pornp., Chantar. & J.Parn. sp. nov.

332DCBBB-E11B-51C6-9F50-5C9E826BDA63

urn:lsid:ipni.org:names:77203519-1

Figs 1 , 2

##### Diagnosis.

*Phyllanthus huamotensis* is one of the most distinct species of *Phyllanthus* in Thailand, easily distinguished by its reddish branchlets and stem, conspicuous reddish venation, especially on the lower leaf surface, red sepals with long fimbriate margins and red capsule with a papillose-puberulous surface. It is most similar to *P. pulcher* Wall. ex Müll.Arg., but differs in its undershrub habit that is up to 30 cm high (*P. pulcher* is a shrub up to 1.5 m high), small sized leaves (2–9 × (2–)3–8 mm) (leaves in *P. pulcher* are 7–28 × 6–17 mm) with conspicuous reddish venation (inconspicuous on both leaf surfaces in *P. pulcher*) and a red capsule with a papillose-puberulous surface (glabrous in *P. pulcher*).

##### Type.

Thailand. Tak, Umpang district, Doi Hua Mot; 16°2.63'N, 98°51.26'E; alt. 901 m; 22 Aug. 2019; *P. Pornpongrungrueng*, *N. Triyutthachai*, *S. Ninkaew & S. Sukcharoen 1287* (***holotype*** KKU; ***isotypes*** BKF, K, QBG, TCD).

##### Description.

Undershrubs up to 30 cm high, branchlets and stem reddish, terete, young branchlets minutely puberulous. *Stipules* triangular-lanceolate, 0.5–1 × 0.3–0.5 mm, glabrous. *Leaves* alternate; petioles 0.4–0.9 mm long, glabrous; lamina broadly ovate, obovate, rounded, broadly elliptic, ovate-oblong, 2–9 × (2–)3–8 mm, subcoriaceous, glabrous on both surfaces, base oblique, cordate, broadly cuneate, truncate, rounded, margin entire, revolute, apex acute, acuminate, rounded; nerves in 4–6 pairs; reticulation reddish, conspicuous, especially on the lower surface. *Flowers* red, unisexual; staminate flowers 2–3(–4) in axillary fascicles in proximal axils; pistillate flower solitary in distal axils. *Bracts* subulate, 0.2–0.3 × 0.1–0.2 mm, glabrous. *Staminate flowers*: pedicel 4–10 mm long, glabrous; sepals 4, red, triangular, rhombic-ovate, lanceolate, 1.5–2 × 1–1.2 mm, glabrous, margin long fimbriate; disc glands 4, reniform; stamens 4, staminal column ca. 0.2 mm long, anthers ca. 0.2 mm long, transversely dehiscent. *Pistillate flowers*: pedicel 7–17 mm long, glabrous; sepals 5–6, red, rhombic-ovate, 1.5–3 × 0.6–1 mm, membranous, glabrous, margin fimbriate; disc glands 5 or 6, free, obovate with truncate apex; ovary superior, ca. 0.7 mm diam., 3-locular, ovules 2 per locule, papillose-puberulous; styles 3, free, ca. 0.1 mm long; stigmas nearly completely bifid, ca. 0.2 mm long, glabrous. *Fruits* capsule, red, 2.5–3 mm diam., papillose-puberulous; pedicel 7–17 mm long. *Seeds* trigonous, brown, 1.5–1.8 × 1.1–1.2 mm, surface transversely striate.

**Figure 1.**
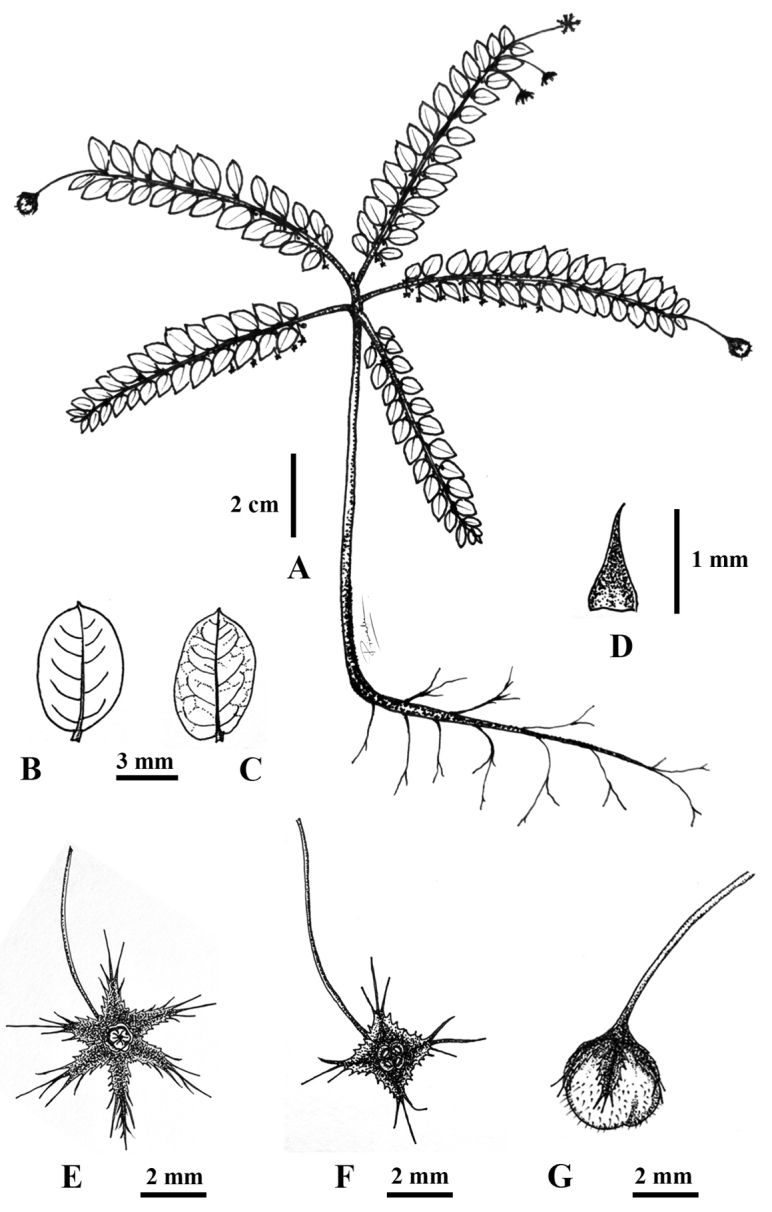
*Phyllanthus huamotensis* Pornp., Chantar. & J.Parn., sp. nov. **A** habit **B, C** leaf shapes (**B** adaxial surface **C** abaxial surface) **D** stipule **E** pistillate flower **F** staminate flower **G** mature capsule. Drawn by Pimwadee Pornpongrungrueng.

**Figure 2.**
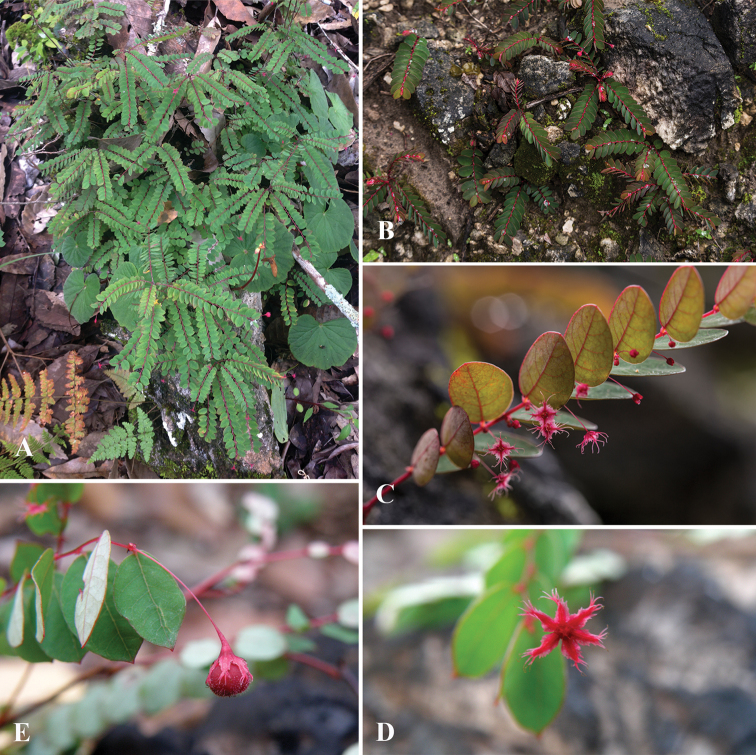
*Phyllanthus huamotensis* Pornp., Chantar. & J.Parn., sp. nov. **A, B** habit **C** branchlet showing axillary fascicle of staminate flowers **D** branchlet showing pistillate flower **E** branchlet showing young red capsule. **A** Photo by Natthawut Triyuttachai **B, C** photos by Suchart Chanhomhual **D, E** photos by Kanokorn Ruengsawang.

##### Phenology.

Flowering and fruiting period is June to December.

##### Habitat and distribution.

This species grows on open limestone hills, at 880–937 m above sea level. Currently, it is known only from the type location Doi Huamot, Tak province in northern Thailand.

##### Conservation status.

The species is only known from the type locality. It should be categorised as Critically endangered [CR, B1ab (iii)] according to the IUCN Red List Criteria and Categories version 3.1 (IUCN 2012). The extent of occurrence is estimated to be less than 20 km^2^ and this species is found in a restricted area on open limestone hills which is a threatened ecosystem.

##### Etymology.

The name of this species is given, based on the location where the plant was first discovered.

##### Vernacular.

Ma Kham Pom Din Huamot.

##### Additional specimens examined.

**Thailand**: Tak, Umpang district, Doi Hua Mot; 15°56.46'N, 98°51.93'E; alt. 937 m; 2 Dec. 2018; *P. Pornpongrungrueng*, *N. Triyutthachai & P. Chantaranothai 1270* (BKF, KKU), ibid.; 15°51.40'N, 98°50.88'E; alt. 882 m; 22 Aug. 2019; *P. Pornpongrungrueng*, *S. Ninkaew*, *S. Sukcharoen & N. Triyutthachai 1285* (BKF, KKU, TCD).

#### Phyllanthus chantaranothaii

Taxon classificationPlantaeMalpighialesPhyllanthaceae

Pornp., J.Parn. & Hodk. sp. nov.

EFB83474-1292-5AAF-8E0A-49BFE5B2673A

urn:lsid:ipni.org:names:77203520-1

Figs 3 , 4

##### Diagnosis.

*Phyllanthus chantaranothaii* is most similar to *P. pulcher*, but differs in its puberulous upper leaf surface with white, simple and dendritic hairs and pistillate flowers that have red, narrowly lanceolate sepals with a white long fimbriate margin, puberulous on the outer side and puberulous pedicel, whereas in *P. pulcher*, the leaf is glabrous on both surfaces and the sepals of the pistillate flower are rhombic-ovate with the upper part greenish and lower part red, glabrous on the outside and glabrous pedicel.

##### Type.

Thailand. Tak, Umpang district, Pa La Ta waterfall, 15°49.14'N, 98°51.37'E, alt. ca. 500 m, 23 Aug. 2019, *P. Pornpongrungrueng*, *S. Ninkaew*, *S. Sukcharoen & N. Triyutthachai 1291* (***holotype*** KKU; ***isotypes*** BKF, K, QBG, TCD).

##### Description.

Small shrubs up to 80 cm high, branchlets terete, young branchlets puberulous with white, simple and dendritic hairs. *Stipules* lanceolate-subulate, 1.4–2 × 0.1–0.4 mm, glabrous. *Leaves* alternate; petioles ca. 0.5 mm long, glabrous; lamina oblong, obovate, 1–2.1 × 0.5–0.8 cm, membranous, upper surface puberulous with white, simple and dendritic hairs, lower surface glabrous, base oblique, margin entire, revolute, apex mucronate; nerves in 4–7 pairs; reticulation inconspicuous on both surfaces. *Flowers* unisexual; staminate flowers 2–3 in axillary fascicles along lower half of the branchlets; pistillate flower solitary in leaf-axils along upper half of the branchlets. *Bracts* subulate, 0.6–1.4 × ca. 0.2 mm, puberulous-glabrous. *Staminate flowers*: pedicel 5–11 mm long, glabrous; sepals 4, red, triangular, rhombic-ovate, 2–3 × ca. 1 mm, glabrous, margin white long fimbriate; disc glands 4, reniform; stamens 4, staminal column ca. 0.2 mm long, anthers ca. 0.2 mm long, transversely dehiscent. *Pistillate flowers*: pedicel 8–11 mm long, puberulous; sepals 6, reddish, narrowly lanceolate, 3–3.5 × 0.5–0.8 mm, outer surface puberulous, margin white long fimbriate; disc glands 6, free, obovate with truncate apex; ovary superior, ca. 1 mm diam., 3-locular, ovules 2 per locule, glabrous or papillose; styles 3, free, ca. 0.1 mm long; stigmas nearly completely bifid, 0.4–0.6 mm long, glabrous. *Fruits* capsule, young capsule white to pale greenish, 2.5–4 mm diam., glabrous or papillose; pedicel 5–13 mm long. *Seeds* trigonous, brown, 1.5–2 × 1–1.3 mm, surface transversely striate.

**Figure 3.**
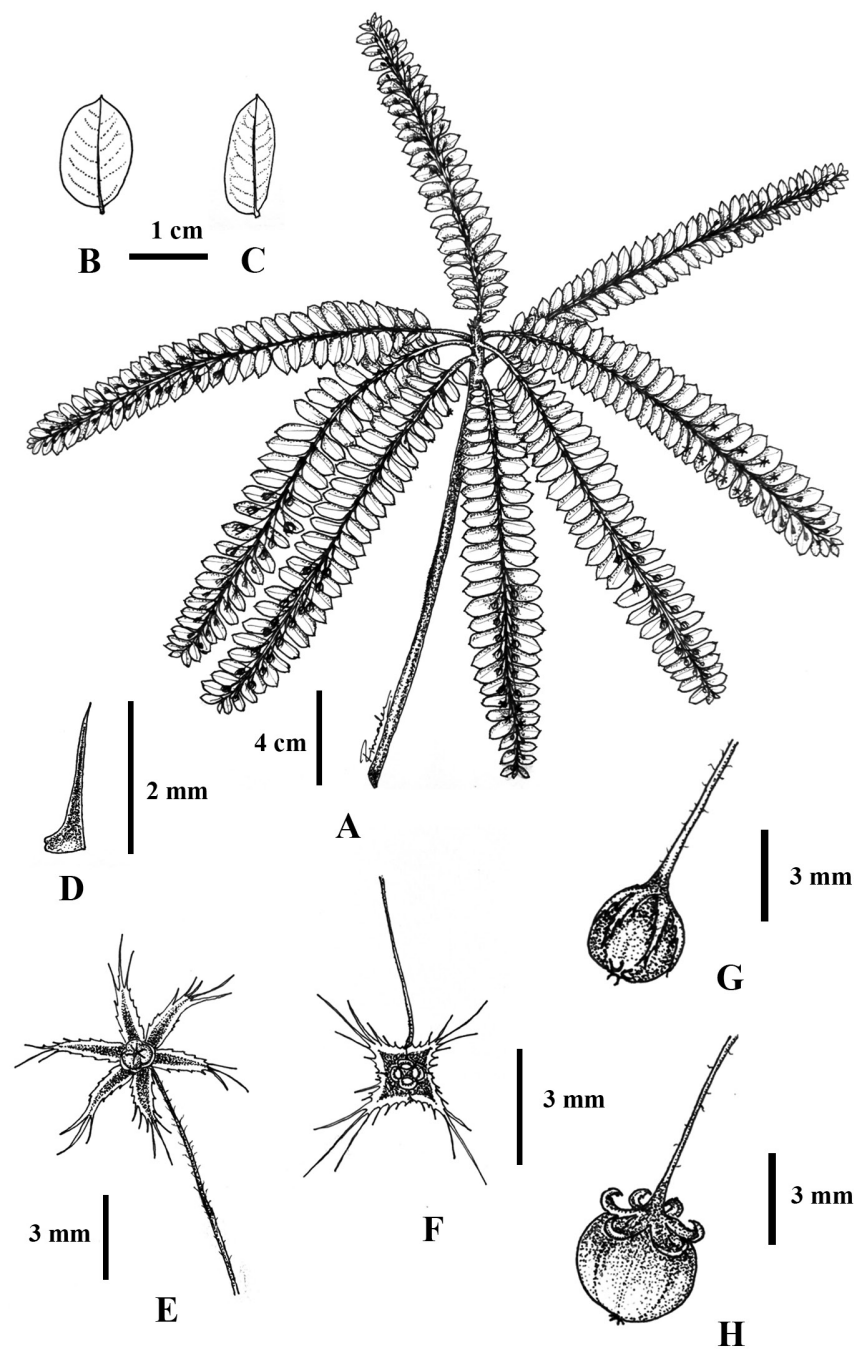
*Phyllanthus chantaranothaii* Pornp., J.Parn. & Hodk., sp. nov. **A** habit **B, C** leaf shapes (**B** adaxial surface **C** abaxial surface) **D** stipule **E** pistillate flower **F** staminate flower **G** young capsule **H** mature capsule. Drawn by Pimwadee Pornpongrungrueng.

**Figure 4.**
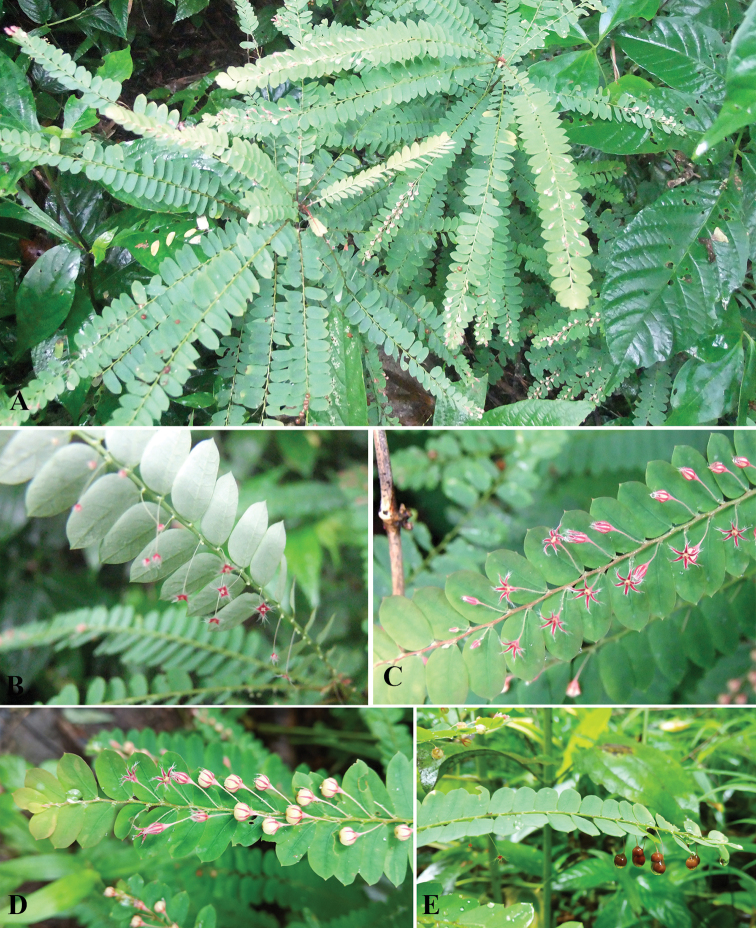
*Phyllanthus chantaranothaii* Pornp., J.Parn. & Hodk., sp. nov. **A** habit **B** branchlet showing axillary fascicle of staminate flowers **C** branchlet showing pistillate flower **D** branchlet showing young capsule **E** branchlet showing mature capsule. **A–C** photos by Natthawut Triyuttachai **D, E** photos by Siriyakorn Sukcharoen.

##### Phenology.

Flowering and fruiting from August to November.

##### Habitat and distribution.

This species grows in mixed deciduous forest, at ca. 500 m elevation. It is currently known from the type location near Pa La Ta waterfall and Doi Huamot, Tak province, Thailand.

##### Conservation status.

As only the type collection, which was collected from mixed deciduous forest, has been investigated in detail, more field exploration in similar habitats in the surrounding areas should be conducted in order to provide a more accurate distribution range of this species. However, based on information that is available to us, this species is preliminarily categorised here as Endangered [EN, B1ab(i, iv)], according to the IUCN Red List Criteria and Categories version 3.1 (IUCN 2012). The extent of occurrence is estimated to be less than 50 km^2^ and, previously, it was found in two locations (Doi Huamot and Pa La Ta waterfall in Umpang district, Tak province), but recently, the extent of occurrence seems to be in decline, because the species has not been found in Doi Huamot since it was first photographed in November 2008.

##### Etymology.

The name of this species honours Prof. Dr. Pranom Chantaranothai for his major contributions to plant taxonomy, in general, but especially for his extensive work on *Phyllanthus* in the Flora of Thailand.

##### Vernacular.

Mayom Noi.

## Discussion

The two species described herein should be classified in Eriococcus (Hassk.) Croizat & Metcalf and Section Eriococcus (Hassk.) Croizat & Metcalf, because they have staminate flowers composed of four sepals with a long fimbriate margin, four stamens with transversely dehiscent anthers and the stigmas in pistillate flowers are free and bifid. These are diagnostic characters of the section Eriococcus which occurs predominantly in mainland Asia, especially in Indochina (Kawakita and Kato 2017; Bouman et al. 2018). Prior to this paper, there were seven species recorded in Thailand that belonged to this section, including *P. elegans* Wall. ex Müll.Arg., *P. gracilipes* (Miq.) Müll.Arg., *P. pulcher*, *P. pulchroides* Beille, *P. sikkimensis* Müll.Arg., *P. sootepensis* and *P. taxodiifolius* Beille (Chantaranothai 2007; Bouman et al. 2018). The two newly described species are most similar to *P. pulcher*, but there are a number of different characters as presented in Table 1. Actually, *P. huamotensis* is one of the most distinct species of *Phyllanthus* in Thailand. It can be easily distinguished by its reddish branchlets and stem, conspicuous reddish venation, especially on the lower leaf surface, red sepals with long fimbriate margin and red capsule with papillose-puberulous surface.

**Table 1.** Comparison of morphological characteristics of *P. huamotensis*, *P. chantaranothaii* and *P. pulcher*.

Characters	*P. huamotensis*	*P. chantaranothaii*	*P. pulcher*
habit	undershrubs up to 30 cm high	small shrubs up to 80 cm high	shrubs up to 1.5 m high
branchlet	young branchlets minutely puberulous with simple hairs	young branchlets puberulous with white, simple and dendritic hairs	young branchlets puberulous with white dendritic hairs
leaf shape	broadly ovate, obovate, rounded, broadly elliptic, ovate-oblong	oblong, obovate	oblong to elliptic
leaf size	2–9 × (2)3–8 mm	10–21 × 5–8 mm	7–28 × 6–17 mm
leaf texture	subcoriaceous	membranous	subcoriaceous
leaf base	oblique, cordate, broadly cuneate, truncate, rounded	oblique	oblique
leaf apex	acute, acuminate, rounded	mucronate	abruptly mucronate
upper leaf surface	glabrous	puberulous with white, simple and dendritic hairs	glabrous
leaf reticulation	conspicuous, especially on lower surface	inconspicuous on both surfaces	inconspicuous on both surfaces
staminate flower arrangement	2–3(4) flowers in axillary fascicle along lower half of the branchlets	2–3 flowers, in axillary fascicle along lower half of the branchlets	2–6 flowers in axillary fascicle in proximal axils
staminate flower pedicel	4–10 mm long, glabrous	5–11 mm long, glabrous	6–15 mm long, glabrous
staminate flower sepal	4, red, triangular, rhombic-ovate, 1.5–2 × 1–1.2 mm, long fimbriate margin	4, red, triangular, rhombic-ovate, 2–3 × 1 mm, long fimbriate margin	(3)4, red, triangular or ovate, 2–3 × 1–1.6 mm, long fimbriate margin
pistillate flower arrangement	solitary in distal axils	solitary in leaf-axils along upper half of the branchlets	solitary in distal axils
pistillate flower pedicel	7–17 mm long, glabrous	8–11 mm long, puberulous	14–25 mm long, glabrous
pistillate flower sepal	5–6, red, rhombic-ovate, 1.5–3 × 0.6–1 mm, glabrous	6, reddish, narrowly lanceolate, 3–3.5 × 0.5–0.8 mm, outer surface puberulous	(5)6, lower part red, upper part greenish, rhombic-ovate, 2–4.5 × 1.1–2.5 mm, glabrous
ovary	papillose-puberulous	glabrous or papillose	glabrous
styles	ca. 0.1 mm long	ca. 0.1 mm long	ca. 0.1 mm long
stigma	ca. 0.2 mm long	0.4–0.6 mm long	0.3–0.4 mm long
capsule	young capsule red, 2.5–3 mm diam., papillose-puberulous	young capsule white to pale greenish, 2.5–4 mm diam., glabrous or papillose	young capsule light greenish-red, ca. 2.5 mm diam., glabrous
seed	1.5–1.8 × 1.1–1.2 mm	1.5–2 × 1–1.3 mm	2–3 × 0.3–0.5 mm

## Supplementary Material

XML Treatment for Phyllanthus huamotensis

XML Treatment for Phyllanthus chantaranothaii
